# Bilateral juvenile temporal arteritis: a case-based review

**DOI:** 10.1007/s00296-024-05624-2

**Published:** 2024-08-24

**Authors:** Joana Marques-Soares, Mª Isabel Garcia-Domingo, Cinthya Báez Leal, Jaume Alijotas-Reig

**Affiliations:** 1 Systemic Autoimmune Diseases Unit, Department of Internal Medicine, Hospital Universitari Vall d’Hebron and Systemic Autoimmune Research Unit, Vall d’Hebron Research Institute, Universitat Autònoma de Barcelona, Passeig Vall d’Hebron 119-29, 08035-Barcelona, Catalonia, Barcelona, Spain; 2 Department of Medicine, Faculty of Medicine, Universitat Autònoma de Barcelona, Barcelona, Spain; 3 Department of General Surgery, Hospital Universitari Mútua Terrassa, Universitat de Barcelona, Terrassa, Spain; 4 Department of Pathology, Hospital Universitari Mútua Terrassa, Universitat de Barcelona, Terrassa, Spain

**Keywords:** Case report, Juvenile temporal arteritis, Vasculitis, Systematic review

## Abstract

Juvenile Temporal Arteritis (JTA) is a rare non-granulomatous vasculitis affecting the superficial temporal arteries, mostly in individuals under 45 years old. It is often misdiagnosed due to its benign nature and the absence of systemic symptoms. Herein, we present a case report of a 40-year-old woman who initially presented with painless nodules in the left temporal area. Following a biopsy, the patient developed additional nodules not only in the same temple but also on the contralateral side. Remarkably, these nodules underwent spontaneous regression without further treatment, highlighting the variability in JTA’s course and distinctive response to intervention. In addition, through a systematic literature review of 43 case reports – 17 with bilateral involvement – we aimed to thoroughly understand the clinical and histopathological findings, diagnostic processes, and treatment responses in JTA, with an emphasis on cases with bilateral involvement. Findings indicate that JTA typically presents as painless or painful temporal nodules, rarely accompanied by other non-specific symptoms, making histopathological examination crucial for accurate diagnosis. Collectively, our work provides the most extensive account of bilateral JTA cases to date. It emphasizes the need for clinical awareness of this condition, contributes valuable data to the limited information available on this rare condition and serves as a stepping-stone for further inquiry. The main takeaway from this review is the variable nature of JTA and the importance of histopathology in diagnosis, which helps clinicians avoid excessive testing and overtreatment and anticipate possible spontaneous resolution.

**Supplementary Information:**

The online version contains supplementary material available at 10.1007/s00296-024-05624-2.

## Introduction

Juvenile temporal arteritis (JTA) is a rare, localised form of non-granulomatous, eosinophilic inflammation confined to the superficial temporal arteries affecting young individuals (from late childhood to early adulthood, usually defined under 45 years of age) first characterised by Lie et al. in 1975 [1]. Notwithstanding initial scepticism [2], the condition is now widely accepted, and most of the published cases present Tomlinson et al. [3] diagnostic criteria suggested in 1994.

Clinically, JTA often presents in a seemingly harmless manner, specifically targeting the branches of the external carotid artery, manifesting as a palpable nodule(s) in the temporal area, which may be non-tender or painful. Key distinguishing factors from other conditions like giant cell arteritis (GCA) include a male predominance, onset in early adulthood, and an absence or minimal presence of symptoms such as polymyalgia, visual disturbances, malaise or fever [4, 5].

Current epidemiological insights indicate that Juvenile Temporal Arteritis (JTA) is exceptionally uncommon, with prevalence estimates under 1 per 1,000,000 individuals [6, 7]. Given its rarity, comprehensive data on the hereditary patterns of JTA is lacking, warranting further scientific investigation to elucidate potential genetic and inheritance patterns, if any.

A hallmark feature in the laboratory assessment of JTA, in contrast to GCA, is the normality of serum acute phase reactants (APR) such as erythrocyte sedimentation rate (ESR) and C-reactive protein (CRP) or other indicators of systemic inflammation (i.e. normocytic anaemia, thrombocytosis, elevated fibrinogen levels, or abnormalities in serum protein electrophoresis (SPEP) like reduced albumin and heightened alpha2 and alpha1 fractions) [5]. Increased serum IgE levels along with hypereosinophilia are common occurrences in JTA patients. In fact, Journeau’s review highlighted that hypereosinophilia was present in one-third of the cases studied [4]. Interestingly, such findings are also observed in two confounder disorders, such as Kimura’s disease (KD) and Angiolymphoid Hyperplasia with Eosinophilia (ALHE) [8, 9].

Similar to GCA, Doppler ultrasound of temporal arteries in JTA displays the halo sign [10]. Inversely, in contrast to GCA, Positron Emission Tomography – Computed Tomography (PET-CT) or CT scans reveal no indications of vasculitis in other arterial regions [11].

Histopathological examination is paramount in distinguishing JTA from other look-alike conditions, such as GCA, KD and ALHE. Characteristic histopathological features observed in JTA include lymphoeosinophilic arteritis or panarteritis accompanied by the presence of intraluminal thrombosis potentially associated with parietal microaneurysmal lesions, as well as a disruption of the internal elastic lamina (IEL) and extensive cellular infiltrate of lymphocytes, eosinophils, and plasma cells in the perivascular tissue. Notably, intimal proliferation (IP) and/or intimal hyperplasia (IH), although also observed in GCA, are also frequently identified in JTA. A significant distinction between JTA and GCA is the absence or merely nominal occurrence of multinucleated giant cells and granulomas in JTA. The presence of peripheral blood eosinophilia (PBE) and varying degrees of perivascular eosinophilic infiltrates and vascular proliferation in some reports has led some authors to consider a nosological overlap between JTA, KD and ALHE. After a thorough review of the available reports of isolated JTA and its simultaneous occurrence with KD or ALHE, Journeau et al. later hypothesised that JTA is primarily an inflammatory vascular disease that initially affects the artery and can extend to neighbouring tissues, leading to the development of lymphoid follicles. On the other hand, the inflammatory infiltrates in KD and ALHE, the inflammatory infiltrates mainly affect the subcutaneous tissue or dermis, respectively, and are primarily perivascular. Other features, such as the presence of fibrinoid necrosis (FN), should prompt alternative diagnosis, including antineutrophil cytoplasmic antibodies (ANCA)-associated vasculitis and Polyarteritis Nodosa. The differential diagnosis for JTA further includes lipomas, sebaceous cysts, dermoid cysts, aneurysms or arteriovenous malformations, as well as Buerger’s disease and cryoglobulinaemia-associated vasculitides^12^–^14^ .

Due to the benign nature of JTA, surgical biopsy often serves both diagnostic and therapeutic purposes without additional pharmacological intervention. However, the aetiology and risk factors of JTA remain elusive. Unlike KD, AHLE and “classical” temporal arteritis (GCA), relapses in JTA are infrequent, but its rarity often leads to misdiagnoses and excessive investigations [4]. Moreover, the medium and long-term follow-up are yet to be clarified, and therapeutic approaches lack standardisation [4].

In the case presented here, a young woman exhibited a unique progression of JTA with sequential bilateral temporal nodules that initially did not respond to surgical intervention. Remarkably, following a biopsy of the initial nodules, which allowed the diagnosis of JTA, contralateral nodules developed, and subsequently all nodules regressed spontaneously without further medical treatment. This post-biopsy behaviour is unusual for JTA, making this case a valuable addition to the literature. Our aim is to provide new insights into the clinical presentation and natural history of the disease as well as a deeper understanding of the potential of JTA for self-resolution, suggesting that remission may occur in some patients without the need for intensive medical intervention. It also emphasises the efficacy of different management strategies, particularly the role of conservative approaches. Such strategies could potentially spare the need for more aggressive treatments, such as corticosteroids (CS) or biologic drugs. These drugs are commonly used for conditions with similar clinical manifestations, such as GCA, which is the main differential diagnosis for JTA.

## Case report

**Fig. 1:**
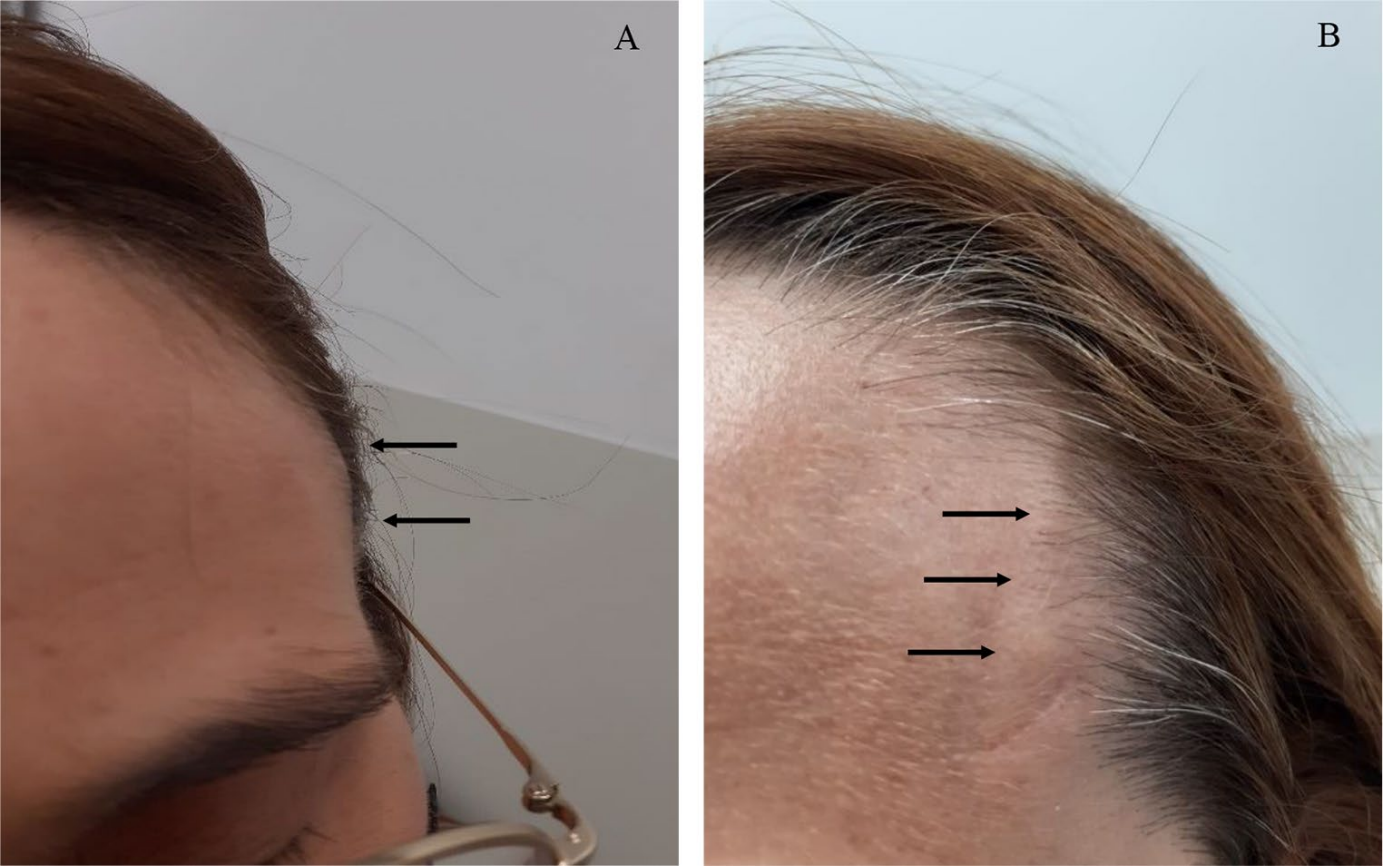
**A**: Clinical presentation of JTA in our patient. Several small-sized painless nodules are located on the superficial left temporal artery, which exemplifies the typical manifestations of JTA. **B**: Reoccurrence of nodules at the site of a previous biopsy on the left temporal artery. These image collectively highlight the characteristic signs of JTA, providing clinicians with a visual reference for identifying it

**Fig. 2:**
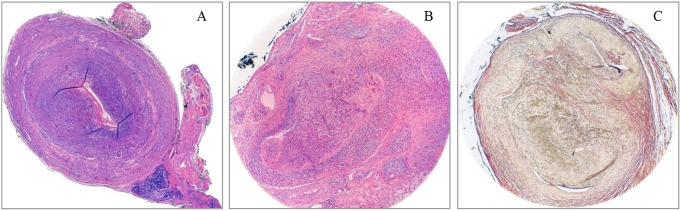
Histopathological findings of the biopsied temporal artery. **A**: Temporal artery. Haematoxylin/Eosin (4X). Cross section of a vascular structure of medium calibre showing architectural distortion with obliteration of the lumen due to inflammatory infiltrate and hyperplasia of the intima layer. **B**: Temporal Artery. Haematoxylin/Eosin (10X). Transmural lymphohistiocytic inflammatory infiltrate with the presence of abundant eosinophils. **C**: Temporal Artery. Verhoeff-Van Gieson staining of elastic fibbers (10x). Special staining demonstrates the loss and disorganization of the elastic fibbers. These visual findings collectively underscore the pathological changes associated with JTA and corroborate the diagnosis based on structural alterations of the vessel

A 40-year-old woman with no significant medical, familial, psycho-social or genetic history was referred to our Unit by her general surgeon for assessment of painless, unilateral nodules on the left temple. Initially presumed to be sebaceous cysts, these nodules had developed gradually over several month. The patient denied headaches, temple allodynia, mandibular pain during mastication, lingual ulcers, visual impairment, or systemic symptoms such as fever, myalgia, weight loss, fatigue, and joint or girdle pain. Physical examination revealed well-circumscribed, painless nodules over the left superficial temporal artery (Fig. 1-A). Notably, the left temporal pulse was attenuated, manifesting pronounced asymmetry compared to the contralateral temporal region. The extra-temple examination was largely unremarkable; no evidence of musculoskeletal, ocular, oral, or cutaneous manifestations was observed. Pulmonary, cardiovascular, and peripheral vascular examinations were normal. The patient’s general physical condition was good, with a body mass index of 21.1 kg/m^2^ and arterial blood pressure recorded at 110/76mmHg, with no appreciable asymmetry between extremities. Abdominal examination revealed no organomegaly, palpable masses, or tenderness. Haematological and biochemical investigations were largely within reference ranges; noteworthy results included a haemoglobin level of 13.7 g/dL, a white blood cell count of 6.5 × 10^9^/L (normal neutrophil, lymphocyte, monocyte, and eosinophil differential counts), and platelet count of 226 × 10^9^/L. APR such as ESR, fibrinogen and CRP were unremarkable. Lactate dehydrogenase (LDH) and SPEP were within expected values. Coagulation tests showed no alterations. Antinuclear antibodies (ANA), ANCA, rheumatoid factor (RF), angiotensin-converting enzyme (ACE) and complement levels (C3 and C4) were negative or normal. Urine sediment exhibited no activity, and proteinuria levels were within normal limits. Chest radiography and electrocardiographic assessments were equally non-contributory. Ultrasonography of the left temple showed hypoechoic regions with some areas showing luminal obliteration, while the axillary artery was unremarkable. Excisional biopsy specimens measuring 1.6 × 0.5 × 0.2 cm and 1.0 × 0.7 × 0.2 cm, respectively, revealed architectural distortion of a medium-sized artery characterised by severe luminal occlusion and intimal thickening. Histopathological analysis confirmed a transmural, dense inflammatory infiltrate predominantly composed of lymphocytes and eosinophils, identifying marked disruptions in the IEL but no GC nor granulomatous formation (Fig. 2A-C). A diagnosis of juvenile temporal arteritis was made.

No treatment was initiated since the nodules initially resolved. During a four-week follow-up, new nodules emerged in both the left (Fig. 1-B) and right temporal regions (not shown), although the patient remained asymptomatic. APR and other biochemical markers persisted stable. Angio-CT scans of the carotid and temporal arteries yielded no significant findings. Although surgical intervention was recommended, the patient chose to undergo a course of CS trial therapy. A new chest X-ray and a QuantiFERON®- TB test were performed, ruling out tuberculosis infection before starting CS. Due to work-related commitments, the patient missed subsequent appointments and neither commenced CS therapy nor underwent surgery. Remarkably, when she returned five months later, the nodules had spontaneously regressed. A twelve-month follow-up confirmed the patient to be asymptomatic with no temporal nodules.

## Methods

### Search strategy

For this systematic review, we conducted a methodical and comprehensive literature search by using a variety of term and keyword combinations, including “juvenile temporal arteritis”, “juvenile”, “temporal”, “arteritis”, “vasculitis” and “young” according to the used database as detailed in provided supplementary material (SM-1). Our search specifically targeted articles written in English and indexed in PubMed (MEDLINE), The Cochrane Library, Scopus, Web of Science (WOS) and Directory of Open Access Journals (DOAJ), covering a publication period from 1975 up to February 2024. The study designs incorporated into our analysis ranged from observational case-control or cohort studies and multicentre studies to case reports and communications, clinical trials, reviews, and systematic reviews. Our exclusion criteria initially focused on excluding articles published before 1975, non-English language publications, animal studies, non-medical studies and research involving subjects under the age of 18.

**Fig. 3:**
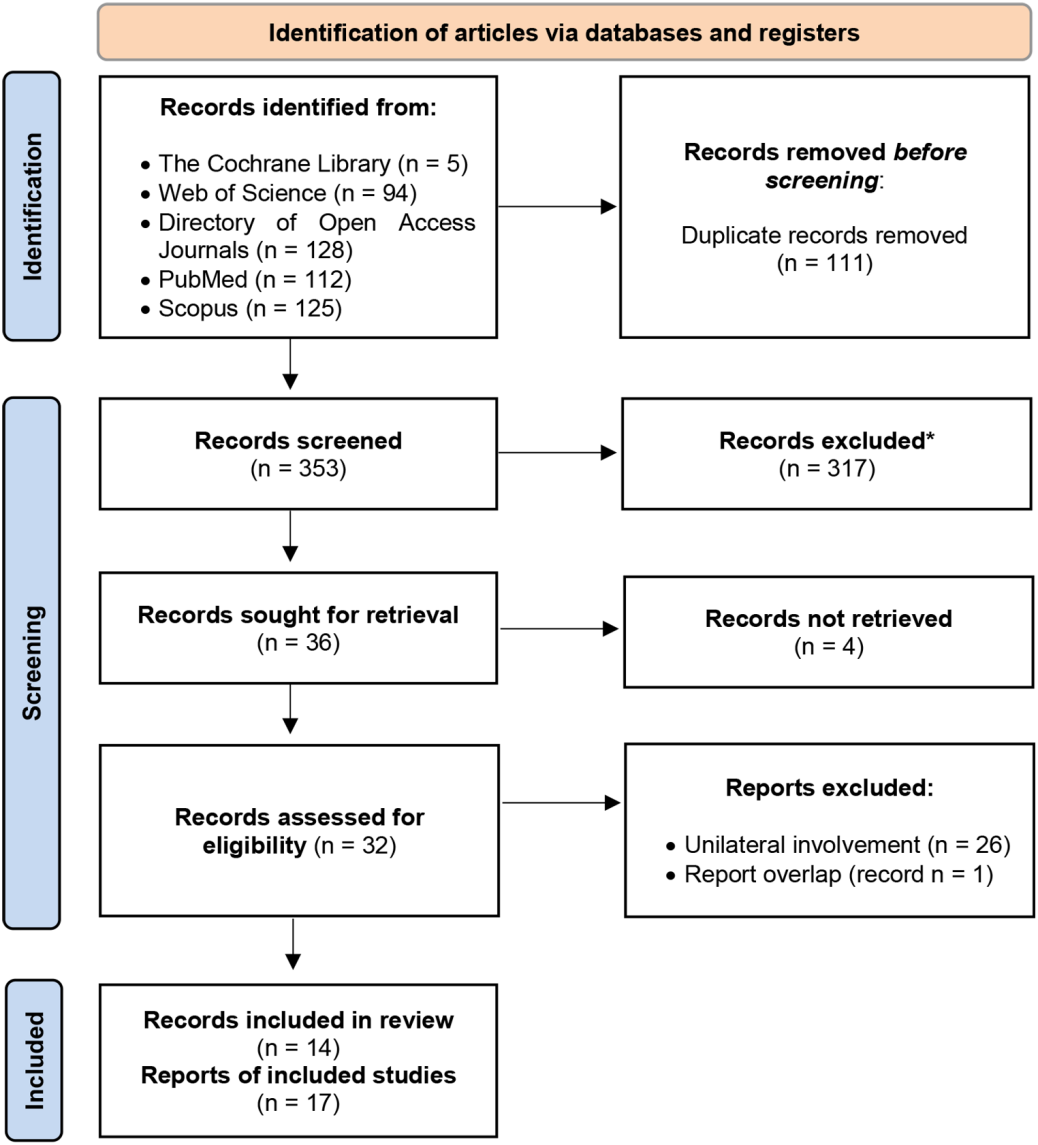
PRISMA Flow Diagram of the record selection process. This flowchart outlines the systematic search and selection method of studies and case reports pertaining to juvenile temporal arteritis (JTA); no automation tools were used in this process. * Enhanced exclusion sensitivity was applied at this stage, excluding records for reasons of irrelevance, deviation from JTA in final diagnoses, incomplete English language publication, and a focus on paediatric patients or patients over the age of 50. Records refer to articles or publications documenting research, while reports refer to detailed accounts of individual cases

A detailed decision process is illustrated in the provided PRISMA flow diagram (Fig. 3). From an initial pool of 464 identified entries, 111 duplicate records were removed. Subsequent assessment of titles and abstracts, if available, led to the exclusion of entries for reasons including lack of retrieval, irrelevance, deviation from JTA in final diagnoses, incomplete English language publication, and a focus on patients over the age of 50. This process ultimately yielded 32 eligible publications [1, 4, 8, 9, 15–42], although one was later excluded [24] as its report was also documented in another case series record [4]. Finally, we focused on bilateral JTA involvement by selecting 14 pertinent articles [4, 16, 18, 20–22, 25, 28, 30, 33, 35, 36, 38, 41] comprising 17 case reports for this review.

## Results and discussion

Although often deemed rare, our review suggests bilateral involvement may be more common than acknowledged. Of the 44 examined cases [1, 4, 8, 9, 15–42], 18 showed bilateral involvement (Table 1), either at diagnosis [4, 16, 18, 20–22, 25, 28, 30, 33, 38, 41] or during follow-up [35, 36].

**Table 1:** Summary of clinical cases on bilateral JTA from 1986 to present. B, bilateral; CBI, initial concurrent bilateral involvement; CS, corticosteroids; EEL, external elastic lamina; EI, eosinophilic infiltration; ESR, Erythrocyte Sedimentation Rate; F, female; FN, fibrinoid necrosis; GC, giant cells; HA, headache; HES, hypereosinophilic syndrome; IF, intimal fibrosis; IH, intimal hyperplasia; IEL, internal elastic lamina; IP, intimal proliferation; L, left; LN, lymph node; M, male; MFN, medial fibrinoid necrosis; MI, mononuclear infiltration; MTX, methotrexate; n, number of adult cases with bilateral involvement; N, normal; NA, information not applicable/not available; PBE, peripheral blood eosinophilia; R, right; RP, Raynaud’s phenomenon; TB; tuberculosis

Reference	*n*/sex/age	Manifestation	Systemic involvement	PBE	↑ IgE	Histopathological findings	Management	Outcome
[18] 1986	1/23/M	Painful nodules (CBI)	None N ESR	+	+	Disruption of IEL. Arteritis with EI. IP. MFN. Thrombosis. Sparse CG. Pseudoaneurysms	Biopsy (L)	Nodule persistence
[33] 1995	1/21/M	Painless nodules (CBI)	None N ESR	NA	NA	Arteritis with EI. IH.	Biopsy (B)	Resolution
[28] 1996	1/39/M	Painless nodules (CBI)	None N ESR	+	-	Obliteration of the arterial lumen. Disruption of IEL. Panarteritis with slight EI. IP. No GC. Microaneurysms.	Biopsy (R). Tocopherol nicotinamide	Nodule persistence
[30] 2004	1/34/M	Painful nodules (CBI), HA	None N ESR	-	NA	Arteritis with MI. Fibrous IP. Thrombosis. No GC	Biopsy (R). Aspirin.	Nodule persistence. HA/tenderness resolution after aspirin
[36] 2006	1/25/M	Painful nodules, HA	Skin rash LN swelling N ESR	+	+	Destruction of arterial wall. EI. Organizing thrombus.	Biopsy (B). CS. Suplatast tosilate	Nodule persistence. HA/tenderness resolution after Suplatast tosilate
[35] 2012	1/31/M	Painful nodules, HA	Fatigue N ESR	-	NA	Disruption of IEL and ELL. Panarteritis with lymphocytic and EI. Fibrous IP. Occlusive thrombus. No GC	Biopsy (L)	R nodule spontaneous resolution; L nodule resolution after biopsy.
[21] 2013	1/35/M	Painful nodules (CBI), HA, sclerosing sialadenitis	Malaise N ESR	-	+	Arteritis with EI. Luminal thrombosis. No GC	Biopsy (B). CS	Nodule persistence. Resolution after CS
[20] 2016	1/39/M	Painless nodules (CBI)	None N ESR	+	+	Disruption of IEL. Panarteritis with lymphocytic, monocytic and EI. IP. No GC	Biopsy (R). CS. MTX	Nodule recurrence (R); nodule persistence (L). Resolution after MTX
[25] 2018	1/23/M	Painless nodules (CBI)	None N ESR	+	NA	IP. EI	Biopsy (B)	Resolution
[4] 2019	4/NA/22–44	Painless nodules (CBI, 3), HA	None	NA	NA	Periarteritis with EI. IH. No GC	Biopsy (NA); colchicine (2)	Resolution (2). R nodule resolution after biopsy, L nodule persistence after biopsy and colchicine (1). R nodule persistence after biopsy, L nodule development; resolution after colchicine (1).
[22] 2019	1/49/F	Painless nodules (CBI), temporal pruritus	None N ESR	+	NA	Obliteration of the arterial lumen. Disruption of IEL. Panarteritis with EI. Fibrous IP. Thrombosis. No GC.	Biopsy (L); CS	Resolution after CS
[38] 2019	1/24/M	Painful nodules (CBI)	LN swelling ESR 26 (*N* < 20)	+	-	Panarteritis with EI. EI in perivascular tissue. Thrombosis. No GC	Biopsy (B); CS (used for HES)	Resolution with CS
[16] 2020	1/36/F	Painful nodules (CBI), visual blurring	ESR 17 (*N* < 15)	+	NA	Obliteration of the arterial lumen. Disruption of IEL. Panarteritis with EI. IH. Microaneurysms. No GC	Biopsy (R)	Nodule persistence
[41] 2021	1/45/M	Painful nodules (CBI), presumed TB-related scleritis (R)	None	-	NA	Disruption of IEL. Fibrinoid degeneration. Arteritis with EI. Perivascular sparse GC and granulomas.	Biopsy (R); CS	Nodule recurrence. Resolution after CS
Current 2024	1/40/F	Painless nodules	None	+	NA	Obliteration of the arterial lumen. Disruption of IEL. Panarteritis with lymphocytic and EI. IH. No CG	Biopsy (L)	Nodule recurrence; posterior spontaneous resolution

Table 2 summarises the main clinical and biological data, treatment modalities, and progression of the cases reviewed. Briefly, the median age of the examined cases was 34.5 years, with a range from 21 to 49 years. A notable difference is observed in the gender distribution, aligning with prior knowledge regarding JTA [4], with males accounting for more than 75% of the patients described [18, 20, 21, 25, 28, 30, 33, 35, 36, 38, 41]. As for presentation, approximately 44.4% of documented cases described the presence of painful nodules [16, 18, 21, 30, 35, 36, 38, 41]. Moreover, a significant 77.8% of cases showed concurrent bilateral involvement (CBI) at diagnosis [4, 16, 18, 20–22, 25, 28, 30, 33, 38, 41]. In contrast to these typical presentations, our patient initially presented with nodules in the left temple; following a biopsy, there was a recurrence on the left side and new nodules developed on both temples. Similarly, Mikami et al. [36] reported a patient who initially presented with right temple nodules that remained after biopsy and subsequently developed nodules on the left temple that remained and worsened following a biopsy on the left side. In the case described by McGeoch et al. [35], the patient exhibited sequential, rather than concurrent, bilateral temporal involvement: the initial right nodule spontaneously resolved before the emergence of a left temple nodule. In a third case described by Journeau et al. [4]. , the initially biopsied right nodule remained, and a left nodule later developed, increasing the overall CBI occurrence to 83.3%.

**Table 2:** Analysis of some of the clinical and histopathological data on bilateral JTA cases. CBI, initial concurrent bilateral involvement; CS, corticosteroid; N/A, not applicable; PBE, peripheral blood eosinophilia

Clinical and biological data	Literature (*n* = 17); references [4, 16, 18, 20–22, 25, 28, 30, 33, 35, 36, 38, 41]	Current (*n* = 1)	Total
Median age (min-max)	34 (21–49)	40	34.5 (21–49)
	n/n (%)	n/n (%)	n/n (%)
Male*	11/14 (78.57%)	0/1 (0%)	11/15 (73.3%)
Painful nodules	8/17 (47%)	0/1 (0%)	8/18 (44.4%)
CBI	14/17 (82.3%)	0/1 (0%)	14/18 (77.8%)
Headache	4/13 (30.8%)	0/1 (0%)	4/14 (28.6%)
Systemic involvement	4/17 (23.5%)	0/1 (0%)	4/18 (22.2%)
Elevated ESR	2/17 (11.8%)	0/1 (0%)	2/18 (11.1%)
PBE	8/12 (66.7%)	1/1 (100%)	9/13 (69.2%)
Elevated IgE	4/6 (66.7%)	N/A	4/6 (66.7%)
Nodule recurrence after biopsy	2/17 (11.8%)	1/1 (100%)	3/18 (16.7%)
Nodule persistence after biopsy	9/17 (52.9%)	0/1 (100%)	9/18 (50%)
CS therapy use	6/17 (35.3%)	0/1 (0%)	6/18 (33.3%)
Resolution after CS therapy	4/6 (66.7%)	N/A	4/6 (66.7%)

*In reference [4], at least one CBI case was identified as female, enabling the gender classification of 15 patients (current case included)

Headaches were comparatively infrequent, documented at 28.6% [4, 21, 30, 35, 36]. Other accompanying local symptoms included TB-related scleritis [41], sclerosing sialadenitis [21] and blurred vision [16] with a concurrent history of Raynaud’s phenomenon, though seemingly unrelated. Minor general symptoms were relatively rare, evidenced in 22.2% [21, 35, 36, 38]. While absent in earlier JTA descriptions and present in only 11.4% of Journeau et al. [4]. recent series, their presence should not exclude a JTA diagnosis.

Concerning APR, only two patients (11.1%) showed marginally elevated ESR values [16, 38], implying limited clinical significance.

PBE was noted in 69% of the combined dataset [16, 18, 20, 22, 25, 28, 36, 38], while elevated IgE levels, though less frequently assessed, were present in 66.7% of the reported instances [18, 20, 21, 36].

Histopathology is crucial for the diagnosis of JTA. In our analysis, eosinophilic infiltration was predominant in 94.4% of cases [4, 16, 18, 20–22, 25, 28, 33, 35, 36, 38, 41], while intimal proliferation or hyperplasia was identified in 77.8% [4, 16, 18, 20, 22, 25, 28, 30, 33, 35], disruption of the internal elastic lamina (IEL) in 44.4% [16, 18, 20, 22, 28, 35, 41] and intraluminal thrombus in 38.9% [18, 21, 22, 30, 35, 36, 38]. Granulomatous lesions or giant cells were anecdotally observed in two cases [18, 41], although described as sparse. These findings highlight the recurrent histopathological features typical of JTA and underline its diagnostic significance.

Contrary to prior literature [8, 38] concerning JTA post-biopsy outcomes, 50% of the considered cases exhibited persistent nodules after biopsy [4, 16, 18, 21, 22, 28, 30, 36], with recurrence in 16.7% [20, 41]. Bilateral involvement can pose a challenge for surgical treatment, as a double biopsy is required and the affected areas are broader, which can lead to cosmetic concerns. Consistent with other reviews [4, 17, 38, 40], no cases progressed to systemic vasculitis, highlighting the benign nature and course of JTA.

Nodule persistence post-biopsy does not suggest a poor prognosis but it certainly compels for a re-evaluation of bilateral JTA management and post-biopsy treatment strategies, although immunosuppressive therapies, unlike in GCA [43], are not the primary approach in JTA. On this subject, when surgical approaches failed or concurrent disorders were present [38], CS therapy was the primary drug choice, given to one-third of patients (33.3%) [20–22, 36, 38, 41], leading to nodule resolution in more than 65% of them [21, 22, 38, 41]. Beyond CS therapy, several other drugs have been sporadically employed, including colchicine [4], tocopherol nicotinamide [28], aspirin [30], suplatast tosilate [36], and methotrexate (MTX) [20], the latter two being used following CS therapy. In one instance [36], suplatast tosilate (a Th2 cytokine inhibitor, able to block both IL-4 and IL-5 synthesis) was introduced following a brief three-day course of prednisolone (25 mg/day), an inadequate duration to assess the efficacy of CS. In another case, when the initial dose of prednisone at 37.5 mg/d, tapered to 10 mg/d over five months, proved insufficient owing to the continuing presence of painful although regressing nodules, MTX was added to the therapeutic regimen as a steroid-sparing agent [20]. This approach eventually facilitated the discontinuation of both CS therapy and subsequently, MTX.

This review provides insights into JTA by critically evaluating the available clinical and histopathological data, being mindful of the implications of bilateral involvement on patient care. It emphasises the importance of well-defined diagnostic criteria and clarifies treatment strategies for JTA. However, limitations include the small sample size, a retrospective design and the focus on English-only articles, which may limit global representativeness. Differences in diagnostic criteria, treatment and follow-up periods, as well as overlapping features in other conditions (i.e., KD, ALHE, HES), may lead to inconsistent conclusions about the diagnosis, management and progression of JTA, further emphasizing the need for precise diagnostic benchmarks.

Future research on JTA should broaden language inclusion, standardise diagnostic criteria and focus on long-term outcomes, including biopsy impact assessments. Longitudinal studies of JTA cases should explore clinical characteristics, demographic correlations, the impact of early intervention and treatment efficacy, particularly comparing unilateral and bilateral cases. Histopathological analysis may provide insights into the aetiology of JTA, while establishing unified follow-up protocols and evaluation of the efficacy of diagnostic procedures will improve the understanding of the clinical course of the disease.

In our case study, the patient reported her satisfaction with the decision to undergo corticosteroid therapy instead of surgical treatment, a decision supported by the subsequent regression of the nodules. Importantly, the actual administration of corticosteroid therapy was not performed, a detail that emphasises the unpredictable nature of JTA and its potential for spontaneous remission. This case contributes to the ongoing debate about the management of JTA and emphasises the need for a tailored treatment approach that takes into account the individual patient’s condition and the variable progression of the disease. It also highlights the importance of considering conservative management in certain cases, which can lead to favourable outcomes without the risks associated with more invasive treatments.

## Conclusions

JTA, a rare vasculitis affecting the extracranial temporal arteries, often presents in individuals under 50 years of age and manifests as painful or painless nodules in the temporal region, typically without systemic symptoms.

Histopathological examination, revealing specific features such as a lymphoeosinophilic infiltrate and the absence of giant cells is essential for an accurate diagnosis and differentiation of JTA from other vasculitic and non-vasculitic disorders. Despite its variable manifestations, JTA patients frequently exhibit normal blood tests and may experience spontaneous remission, highlighting the need for discerning clinical evaluation to avoid unnecessary interventions.

The prevalence of bilateral presentation in JTA cases further emphasises the need for careful and ongoing assessments from initial diagnosis through subsequent follow-up to avoid unnecessary testing and treatments and to be alert to the possibility of spontaneous remission in the clinical course of JTA. Surgical procedures and corticosteroids are the primary treatment modalities, with occasional use of methotrexate based on anecdotal evidence.

A deeper exploration of the implications of bilateral JTA is crucial to improving patient care and enhancing outcomes.

## Electronic supplementary material

Below is the link to the electronic supplementary material.

Supplementary Material 1

Supplementary Material 2
