# Supplementary material for: Bilateral juvenile temporal arteritis: a case-based review
Source: Rheumatol Int. 2024 Aug 24;44(10):2253–61. doi: 10.1007/s00296-024-05624-2 (PMC11393210; doi:10.1007/s00296-024-05624-2)
Supplement: Supplementary file 1 — Supplementary Material 1 [file 296_2024_5624_MOESM1_ESM.docx]

**SUPPLEMENTARY MATERIAL 1**

**Review Search Strings**

| Search Component | Description |
| --- | --- |
| Databases Searched | PubMed/MEDLINE, Scopus, The Cochrane Library, Web of Science, Directory of Open Access Journals |
| Search Strings | - PubMed/MEDLINE: ((("temporal arteritis" AND "young")) OR ("juvenile temporal arteritis")) OR ("temporal" AND "young" AND "vasculitis")   Filters applied: English, from 1975/1/1 - 2024/2/29   - Scopus: ALL ( * {juvenile temporal arteritis} * ) AND ALL ( * {temporal arteritis} * OR *young* ) AND PUBYEAR > 1974 AND ( LIMIT-TO ( LANGUAGE , "English" ) ) - The Cochrane Library: ((("temporal arteritis" AND "young")) OR ("juvenile temporal arteritis")) OR ("temporal" AND "young" AND "vasculitis") in All Text - (Word variations have been searched); Timespan: 1975-01-01 to 2024-02-29 (Publication Date) - Web of Science: ((ALL=("temporal arteritis" AND "young")) OR ALL=("juvenile temporal arteritis")) OR ALL=("temporal" AND "young" AND "vasculitis");   Filters applied: Languages: English; Timespan: 1975-01-01 to 2024-02-29 (Publication Date).   - Directory of Open Access Journals: "temporal vasculitis" OR "juvenile temporal arteritis" OR "temporal arteritis"; Timespan (year): 1975- 2024. |

Supplementary Material 1. Search Strategy Overview. A concise representation of the search strategy employed for the systematic review, outlining the databases used, the terms searched and for clarity, specific filters applied.
