# Supplementary material for: Bilateral juvenile temporal arteritis: a case-based review
Source: Rheumatol Int. 2024 Aug 24;44(10):2253–61. doi: 10.1007/s00296-024-05624-2 (PMC11393210; doi:10.1007/s00296-024-05624-2)
Supplement: Supplementary file 2 — Supplementary Material 2 [file 296_2024_5624_MOESM2_ESM.docx]

Jaume Alijotas-Reig MD, PhD, MSc; Joana Marques-Soares MD

Systemic Autoimmune Diseases Unit. Department of Internal Medicine. Vall d’Hebron University Hospital

Passeig Vall d’Hebron 119-29. 08035-Barcelona, Catalonia, Spain.

March 7^th^, 2024

Dear Dr Armen Y. Gasparyan,

Please find enclosed the manuscript entitled **“Juvenile Temporal Arteritis: Case-Based Review”**, which we would like to have considered for publication in the *Case-Based Reviews* section of Rheumatology International.

Our manuscript presents a unique case of Juvenile Temporal Arteritis (JTA) in a 40-year-old patient, complemented by an extensive literature review focusing on its bilateral manifestation. The framework of our study is the need to elucidate the clinical trajectory of JTA, a non-granulomatous vasculitis, and to enhance the understanding of its bilateral occurrence, about which little is currently known. In our work, we detail the patient's experience of developing painless nodules that underwent spontaneous regression, an intriguing aspect of JTA's clinical behaviour. We extended our investigation to include a systematic literature review of 41 JTA cases, with a specific lens on the 18 cases presenting bilateral involvement. Our findings emphasise the clinical and pathological heterogeneity of JTA and the essential role of histopathological examination in preventing misdiagnosis. Given the benign yet often misinterpreted nature of JTA, our review offers critical insights into its diagnosis and management. This research offers vital insights for the readership of Rheumatology International, aligning with the journal’s dedication to publishing significant research with broad implications. It contributes crucial knowledge on bilateral JTA fostering much-needed clinical awareness of this rare condition.

Please find that our submission adheres to all journal-specific requirements and ethical standards. The corresponding author is Dr Jaume Alijotas-Reig, available for any further correspondence at jaume.alijotas@vallhebron.cat.

We confirm that this manuscript has not been published, submitted, nor is it currently under consideration for publication elsewhere. All authors have approved the manuscript and agree to its submission to Rheumatology International.

The authors also state that they have no commercial or other interest that may have influenced the preparation and results of this manuscript.

We thank you in advance for your kind attention and consideration of this manuscript.

Sincerely,

Jaume Alijotas-Reig MD, PhD, MSc and Joana Marques-Soares MD on behalf of all authors.
